# Supplementary material for: Modulation of Biophysical Properties of Nucleocapsid Protein in the Mutant Spectrum of SARS-CoV-2
Source: bioRxiv. 2024 Mar 22:2023.11.21.568093. Originally published 2023 Nov 22. Preprint. [Version 2] doi: 10.1101/2023.11.21.568093 (PMC10690151; doi:10.1101/2023.11.21.568093)
Supplement: Supplement 1 [file media-1.pdf]

Supplementary Figure S3:

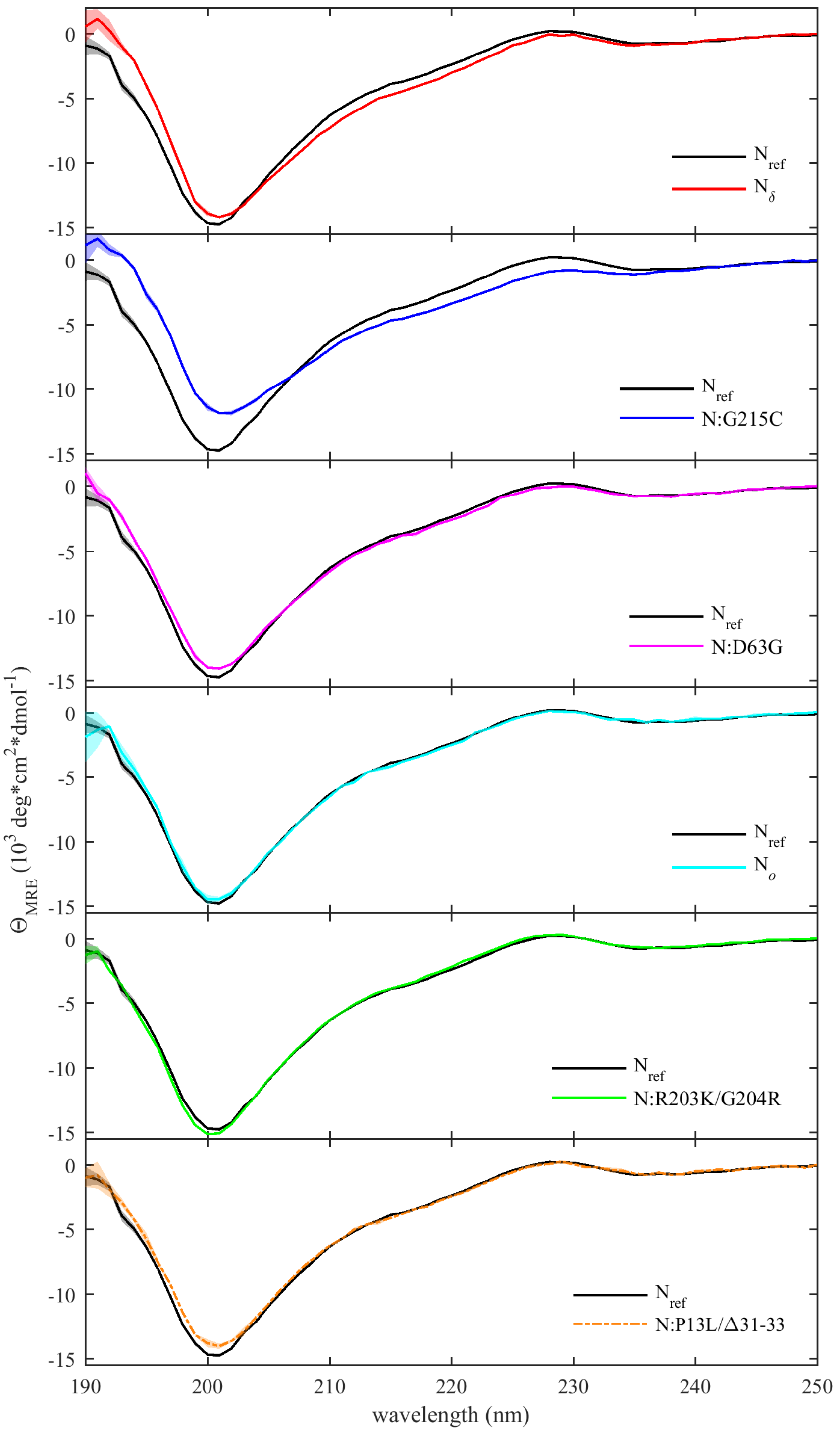

Figure S3. Individual comparison of CD spectra. The data from **Figure 4C** are reproduced and plotted in comparison with N_{ref}. Standard deviations from three acquired spectra are depicted as shaded bands.
