# Supplementary material for: Modulation of Biophysical Properties of Nucleocapsid Protein in the Mutant Spectrum of SARS-CoV-2
Source: bioRxiv. 2024 Mar 22:2023.11.21.568093. Originally published 2023 Nov 22. Preprint. [Version 2] doi: 10.1101/2023.11.21.568093 (PMC10690151; doi:10.1101/2023.11.21.568093)
Supplement: Supplement 4 [file media-4.pdf]

Supplementary Figure S6:

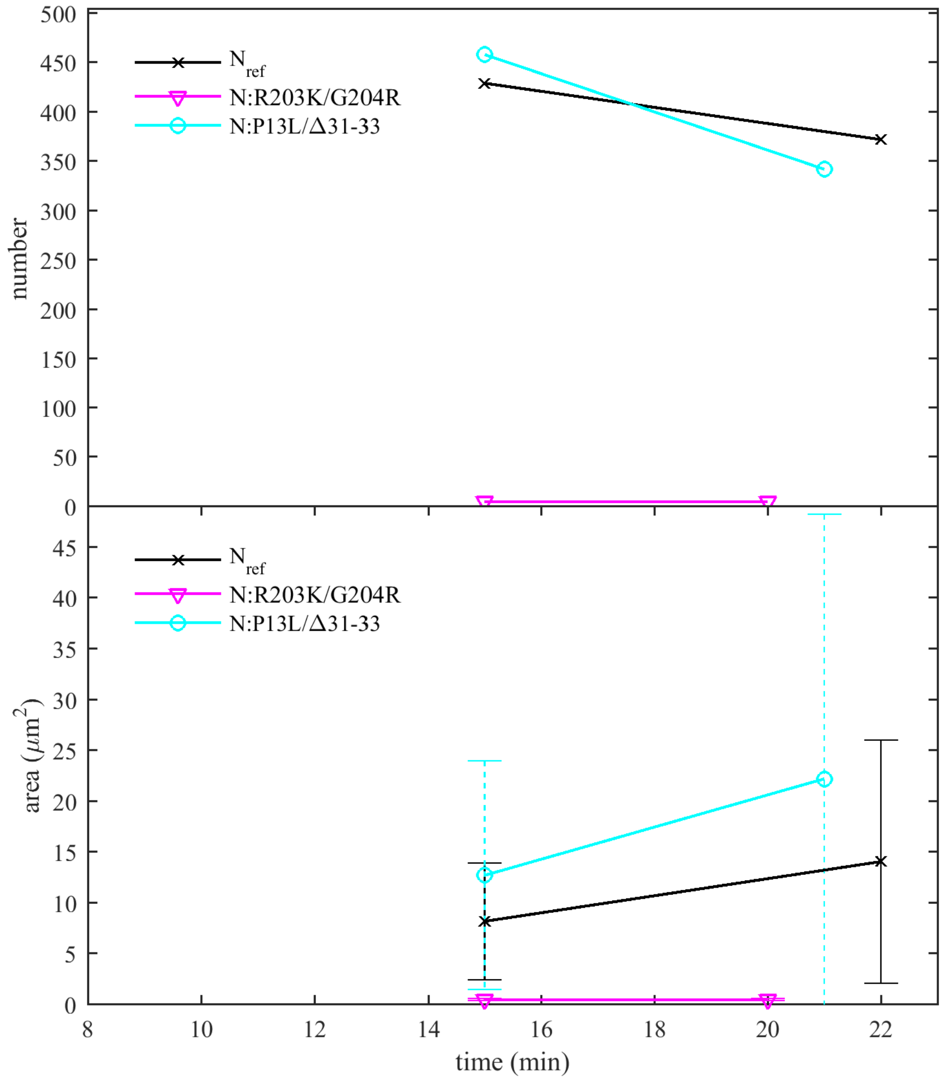

Figure S6. Comparison of droplet area after LLPS at two points in time. Similar to **Figure 7**, images of LLPS were recorded for N_{ref}, N:R203K/G204R, and N:P13L/Δ31-33 at two time-points for the same sample. The upper plot shows droplet numbers. The lower plot shows mean and standard deviations of the droplet area. Images and histograms for the early time points and the later time point of N:P13L/Δ31-33 are shown in **Figure 7** and **Supplementary Figure S4**, respectively.
