# Supplementary material for: Modulation of Biophysical Properties of Nucleocapsid Protein in the Mutant Spectrum of SARS-CoV-2
Source: bioRxiv. 2024 Mar 22:2023.11.21.568093. Originally published 2023 Nov 22. Preprint. [Version 2] doi: 10.1101/2023.11.21.568093 (PMC10690151; doi:10.1101/2023.11.21.568093)
Supplement: Supplement 5 [file media-5.pdf]

Supplementary Figure S7:

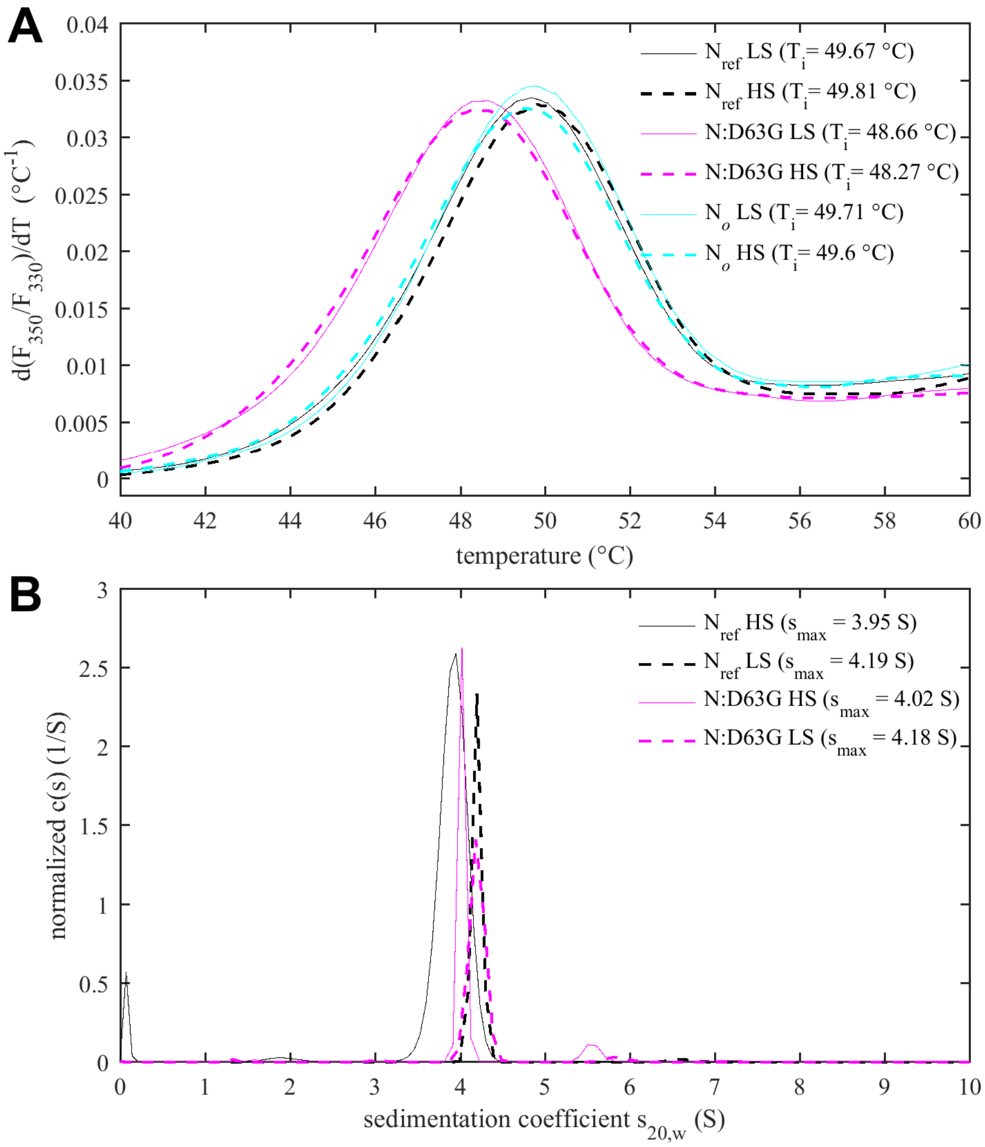

Figure S7. Comparison of solution state of N-protein species in low-salt and high-salt buffer. Protein preparations were dialyzed in either high-salt buffer (HS) consisting of 20mM HEPES, 150mM NaCl, pH 7.5, or low-salt buffer (LS) consisting of 10.1 mM Na_2PO_4, 1.8 mM KH_2PO_4, 2.7 mM KCl, 10 mM NaCl, pH 7.4. (**A**) DSF experiments show no significant shift in T_i for the same protein species in LS or HS buffer. (**B**) SV-AUC exhibit sedimentation coefficient distributions with peak *s*-values increased by ≈5% in LS buffer relative to HS buffer. This apparent change is negligible compared to the ≈60-90% increase in sedimentation coefficients from altered oligomeric states observed for N:G215C and Nδ (**Figure 5A**).
