# Supplementary material for: Modulation of Biophysical Properties of Nucleocapsid Protein in the Mutant Spectrum of SARS-CoV-2
Source: bioRxiv. 2024 Mar 22:2023.11.21.568093. Originally published 2023 Nov 22. Preprint. [Version 2] doi: 10.1101/2023.11.21.568093 (PMC10690151; doi:10.1101/2023.11.21.568093)
Supplement: Supplement 6 [file media-6.pdf]

Supplementary Figure S1:

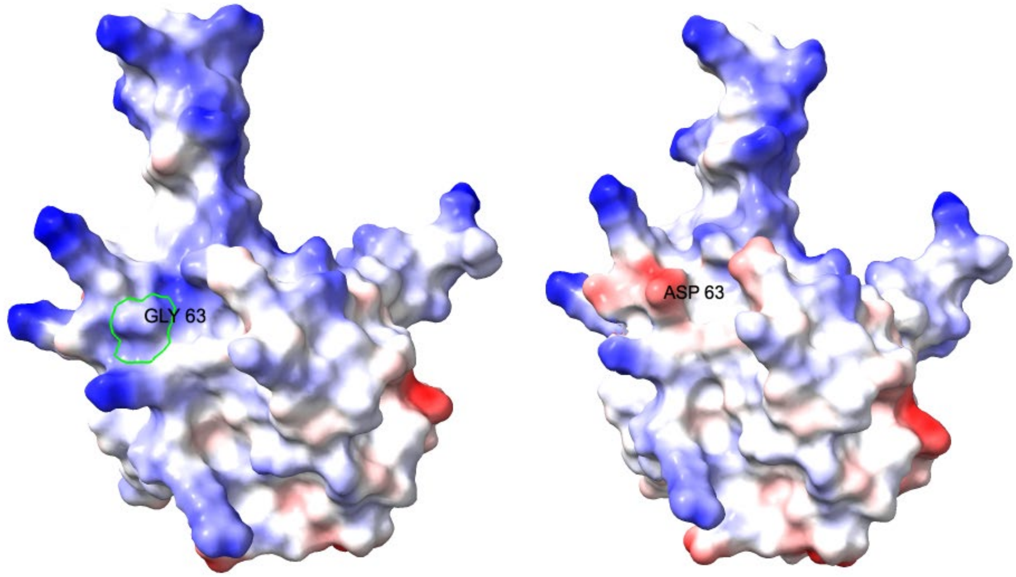

Figure S1. Structural comparison of N:D63G mutant and ancestral N-protein. Structures are predicted using ColabFold for N:D63G (left) and the ancestral protein (right).
