# Supplementary material for: Modulation of Biophysical Properties of Nucleocapsid Protein in the Mutant Spectrum of SARS-CoV-2
Source: bioRxiv. 2024 Mar 22:2023.11.21.568093. Originally published 2023 Nov 22. Preprint. [Version 2] doi: 10.1101/2023.11.21.568093 (PMC10690151; doi:10.1101/2023.11.21.568093)
Supplement: Supplement 7 [file media-7.pdf]

Supplementary Figure S2:

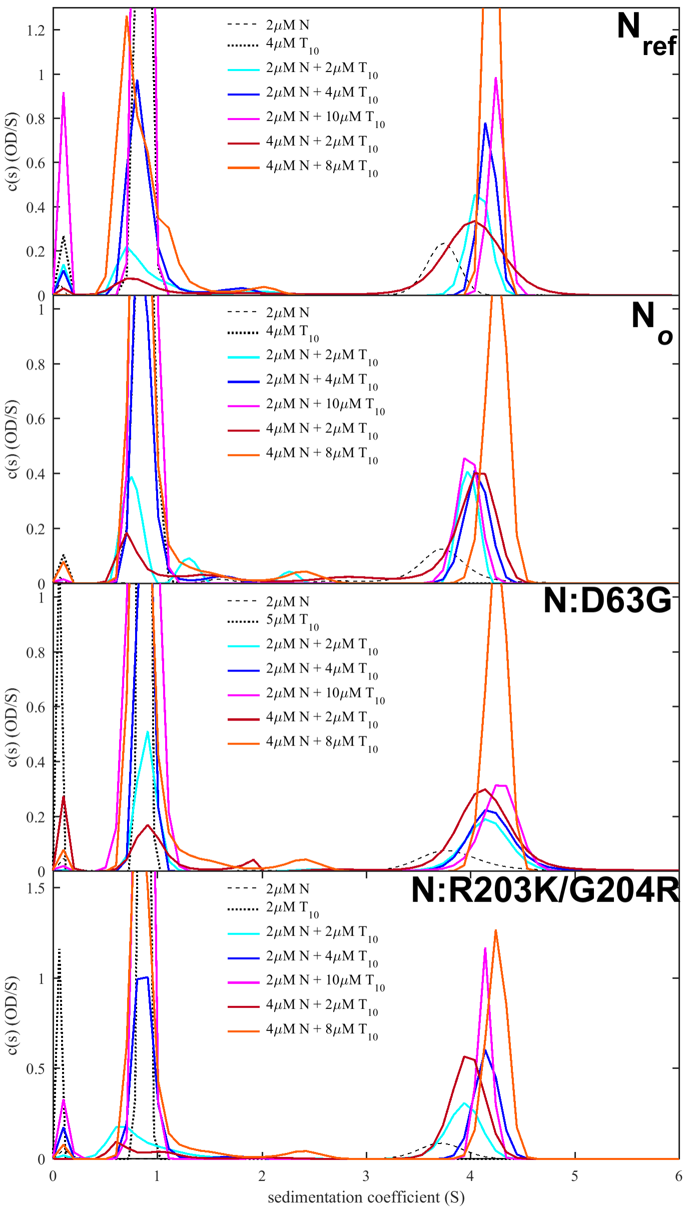

Figure S2. N-protein affinity for binding nucleic acids probed by SV-AUC of N-protein mixtures with oligonucleotide T_{10}. T_{10} can occupy the NA binding grove of the NTD of N-protein, but does not permit multi-valent binding. Titration series of N-protein with T_{10} allows separation of concentration-dependent populations of free and bound/co-migrating T_{10} in the mixtures. This provides the basis for the determination of equilibrium binding constants through non-linear regression of the isotherm of signal-weighted average sedimentation coefficients using a two-site binding model of T_{10} to N-protein dimers. Best-fit K_D-values and 95% confidence intervals are 1.1 [0.8 – 1.6] µM for N_{ref}, 2.8 [1.6 – 4.9] µM for N_o, 2.4 [1.1 – 5.0] µM for N:D63G, and 1.3 [0.9 – 1.9] µM for N:R203K/G204R, respectively. SV-AUC experiments were carried out in buffer HS. Similarly, no significant difference was measured in binding affinity between N_{ref} and N:D63G was observed in buffer LS.
