# Supplementary material for: Modulation of Biophysical Properties of Nucleocapsid Protein in the Mutant Spectrum of SARS-CoV-2
Source: bioRxiv. 2023 Nov 22:2023.11.21.568093. Preprint. [Version 1] doi: 10.1101/2023.11.21.568093 (PMC10690151; doi:10.1101/2023.11.21.568093)
Supplement: Supplementary file 1 [file media-3.pdf]

Supplementary Figure S3:

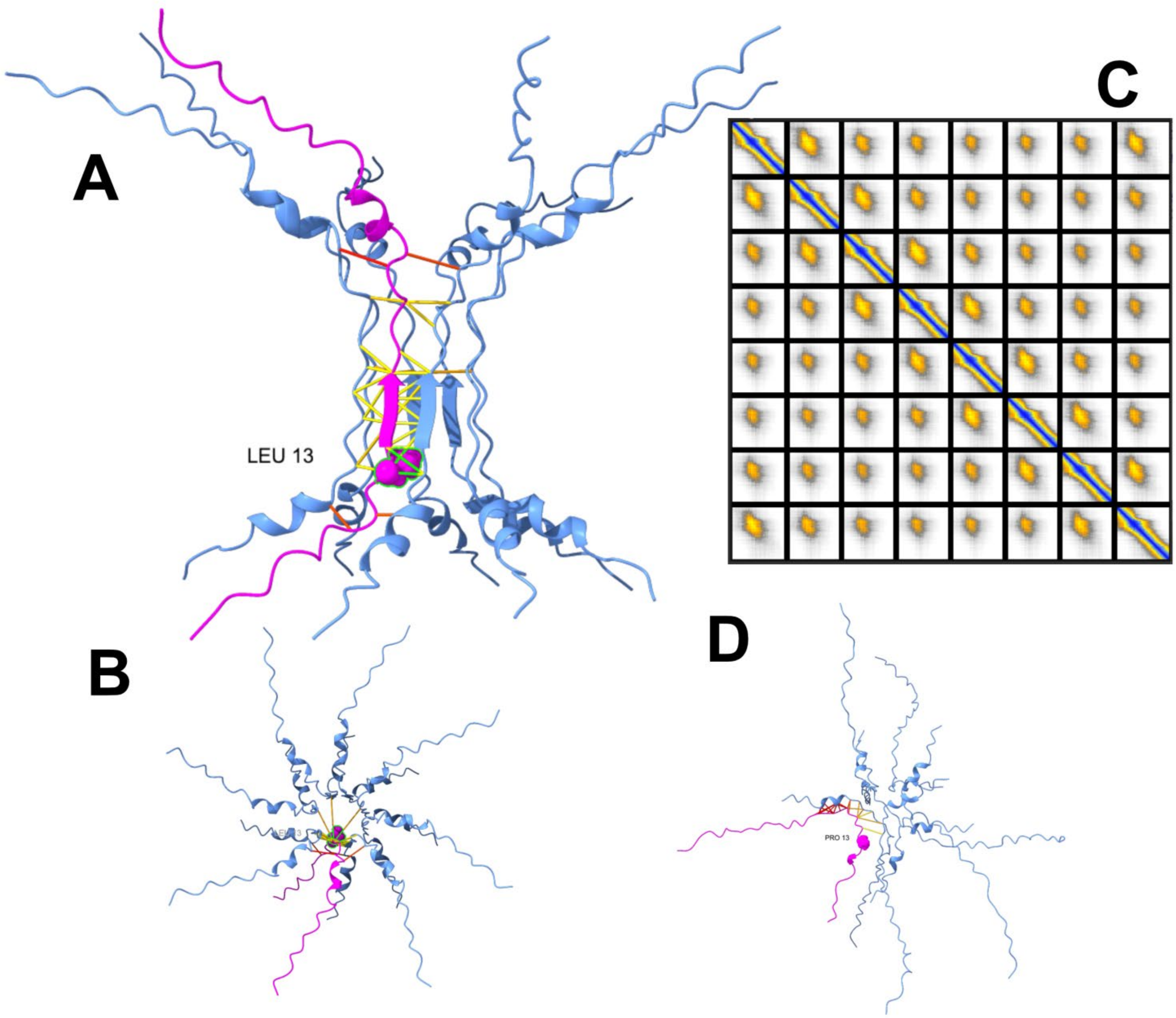

Figure S3. Structural prediction of Omicron N-arm self-interactions. (A) Best ColabFold prediction of eight Omicron N-arm (1:44) peptides with P13L and Δ31-33 mutations. For one chain shown in magenta, atoms of 13L are depicted and labeled, and contacts of this chain within 3.5 Å are color-coded by confidence. (B) Top view of (A). (C) Predicted alignment error (PAE) map showing symmetry and confidence of predicted interactions. (D) Best analogous prediction of ancestral N-arm interactions, highlighting the absence of order.
